# Longitudinal Pain Medication Use Among US Older Adults After a Hip Fracture

**DOI:** 10.1016/j.jamda.2025.105922

**Published:** 2025-10-24

**Authors:** Lexie R. Grove, Andrew R. Zullo, Daniel A. Harris, Oshin Miranda, Richa Joshi, Meghan A. Cupp, Francesca L. Beaudoin, Melissa R. Riester, Kaleen N. Hayes

**Affiliations:** a Center for Gerontology and Healthcare Research, Brown University School of Public Health, Providence, RI, USA; b Department of Health Services, Policy, and Practice, Brown University School of Public Health, Providence, RI, USA; c Department of Epidemiology, Brown University School of Public Health, Providence, RI, USA; d Department of Epidemiology, University of Delaware, Newark, DE, USA

**Keywords:** Pain management, hip fracture, skilled nursing facilities, gabapentinoids, opioids, geriatrics

## Abstract

**Objectives:**

To describe longitudinal pain medication use, including gabapentinoid-opioid co-prescribing, among older adults discharged to a skilled nursing facility (SNF) following a hip fracture.

**Design:**

Retrospective cohort study using Medicare claims linked to medication dispensing records from a large commercial SNF pharmacy.

**Setting and Participants:**

Older Medicare fee-for-service beneficiaries hospitalized for a hip fracture between 2012 and 2018 who were discharged to an SNF.

**Methods:**

We followed beneficiaries from the first day in an SNF up to 100 days to describe opioid, nonopioid analgesic, and gabapentinoid medication regimens dispensed overall and stratified by time since SNF admission, race/ethnicity, and sex.

**Results:**

We identified 88,433 eligible and matched individuals [average age at SNF entry = 84.8 (SD = 8.05) years, 76.7% female]. Of these, 83.7% received any pain medication in the 100 days after SNF admission. The most prevalent pain medication regimen was hydrocodone plus acetaminophen (16.7% prevalence overall), followed by oxycodone plus acetaminophen (11.2%). Dispensing of most medication regimens, particularly opioid-based regimens, declined by more than 50% by day 30 after SNF admission, with oxycodone-based regimens declining more so than hydrocodone-based ones. However, by days 60 to 100, an increase in use of several opioid-based regimens was observed. Among the 6671 individuals prescribed a gabapentinoid in the first 15 days after hospitalization, 91% were co-prescribed an opioid regardless of prior gabapentinoid use before the hip fracture. Prevalence of any pain medication dispensing was similar between sexes and race/ethnicity subgroups, but medication regimens differed by race/ethnicity.

**Conclusions and Implications:**

Most older adults received pain medication after hip fracture, and multimodal opioid-containing regimens were most common. Most individuals who received a gabapentinoid immediately after SNF admission were co-prescribed an opioid. These findings suggest a need for closer medication management in SNFs to ensure adequate pain control after hip fracture while minimizing potentially harmful analgesic combinations.

In the United States, approximately 300,000 older adults are hospitalized for hip fractures each year, with an estimated 70% requiring rehabilitation in skilled nursing facilities (SNFs) and other institutional post-acute care (PAC) settings.^1–4^ Effective pain management in the SNF setting is crucial for facilitating recovery and improving outcomes after hip fracture. Adequate pain control, including use of pain medications, may enhance mobility, increase engagement in physical therapy, and decrease the risk of developing chronic pain and long-term functional impairment.^5–7^

However, the potential benefits of pain medications for hip fracture management must be balanced against their risks. The possible harms of opioid use among older adults are well established and include increased risk of falls and related injuries.^8^ In light of these risks, current clinical practice guidelines recommend use of multimodal pain control regimens that combine multiple drug classes with the goal of reducing opioid use.^9–12^ Potentially because of calls to decrease opioid prescribing, clinicians are increasingly prescribing gabapentinoids to older adults following surgery,^13^ despite mixed evidence on the effectiveness of gabapentinoids in controlling pain and decreasing opioid use.^14,15^ Concomitant use of gabapentinoids and opioids has also risen in recent years and is associated with increased risk of respiratory depression, opioid overdose, and death.^16–18^

Despite the importance of balancing the benefits and risks of pain medication use among older adults, limited evidence is available to inform clinicians’ selection of specific pain management regimens for hip fracture after hospital discharge.^19^ The extent to which guideline-concordant multimodal regimens are currently used after SNF entry is unknown, as is the prevalence of specific pain medication regimens. Such information is needed to inform the design of future comparative effectiveness studies in the SNF PAC setting. However, to date, data limitations have precluded research on medication use in SNFs; medication dispensings in SNFs are unobservable in Medicare claims data due to consolidated payment structures.^20^

In this descriptive study, we created a novel dataset leveraging long-term care pharmacy data and Medicare claims to describe pain medication dispensing among older adults discharged to an SNF following a hip fracture hospitalization. We also assessed the prevalence of opioid-gabapentinoid co-prescribing given the potential for significant harm from drug-drug interactions in this older adult population.^21^

## Methods

### Data Sources

We used 2011–2018 Medicare enrollment and claims data and other administrative data sources. We identified medication dispensings using Medicare Part D claims and pharmacy dispensing data from a large comercial SNF pharmacy, Omnicare, that provides services for more than 1.4 million residents across thousands of long-term care and assisted living facilities in the United States.^22^ Omnicare dispensing records provided information on medication dispensings during periods in which medications were not eligible for Part D reimbursement (ie, during SNF stays, where payment for medications is bundled through Part A), as well as over-the-counter medication use. The Medicare Beneficiary Summary File provided detailed demographic information and Medicare and Medicaid plan enrollment information. The Medicare Provider Analysis and Review File (MedPAR) was used to identify hospitalization dates, hospitalization characteristics, and diagnoses. Dates of entry and discharge to PAC were obtained from the Residential History File, which uses a combination of MedPAR records, other Medicare data, and Minimum Data Set (MDS) clinical assessments to identify transitions of care.^23^ Pain severity/frequency was ascertained using MDS assessments. This study received approval from the Brown University Institutional Review Board, and informed consent was waived due to the use of deidentified administrative data.

### Study Population and Data Linkage

Linkage occurred in a multistep process. First, individuals with a record in Omnicare (2012–2018) were matched deterministically to Medicare beneficiary records using first name, last name, date of birth, and gender. Deterministic linkage was conducted by General Dynamics Information Technology, creating a crosswalk of unique, encrypted identifiers by which to link eligible Medicare beneficiaries to the Omnicare records. Of 4,934,037 eligible individuals in Omnicare, 3,453,836 (70%) were successfully matched on all 4 dimensions to a Medicare beneficiary. Next, the list of matched beneficiaries was restricted to a finalized cohort of 88,433 unique Medicare beneficiaries who were hospitalized for hip fracture, created through the following steps.

**Fig. 1.**
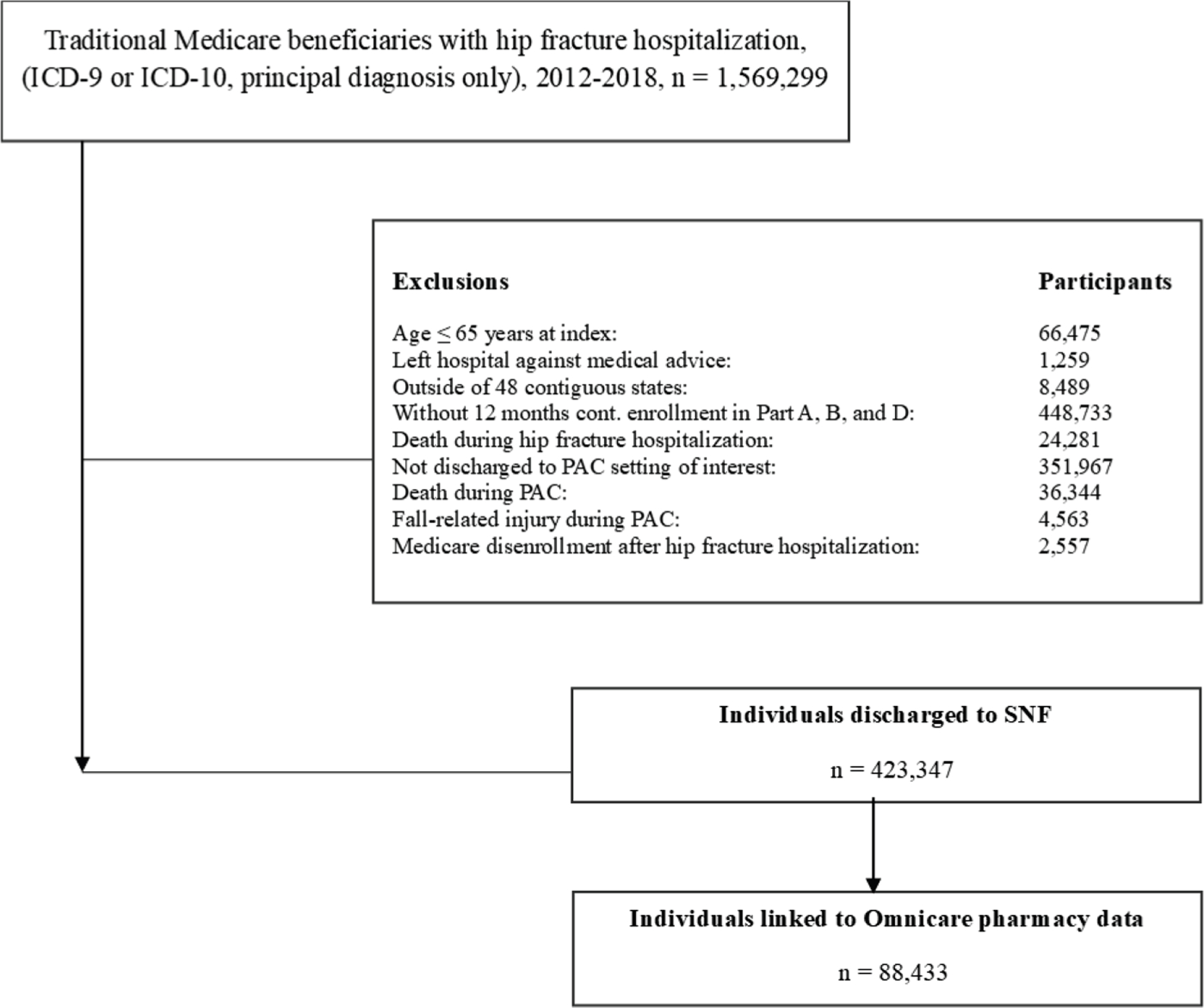
Flow chart of cohort definition. ICD, International Classification of Diseases.

1. We first identified all Medicare beneficiaries with a principal diagnosis of hip fracture on an inpatient claim (codes available in Supplementary Table 1) and then subsequently discharged to an SNF for PAC between January 1, 2012, and December 31, 2018, selecting the first hospitalization during the study period.
2. We then restricted to beneficiaries who were aged ≥65 years as of the hospitalization admission.
3. We then excluded those who were not continuously enrolled in Medicare Parts A, B, and D for at least 12 months before their hip fracture hospitalization; resided outside of the 48 contiguous states; or died during the hospitalization. To examine longitudinal pain medication use among individuals who completed SNF care, we also excluded individuals who died during their SNF stay, disenrolled from Medicare, or had a fall-related injury while in the SNF (cohort flow diagram shown in Figure 1).

### Pain Medication Dispensings and Follow-up

We described pain medication regimens from the date of SNF admission until censoring [death after SNF discharge, end of study data (December 31, 2018), or 100 days after SNF admission]. Patients continued to be followed after SNF discharge using Medicare Part D claims. The full list of eligible pain medications is provided in Supplementary Table 2 and includes acetaminophen (APAP), nonsteroidal anti-inflammatory drugs (NSAIDs), opioids, gabapentinoids, and muscle relaxants (ie, cyclobenzaprine, baclofen). All forms of pain medications were eligible for inclusion, although parenteral forms (eg, infusions) may not have been reliably captured through Omnicare and Part D claims as these formulations are often administered in outpatient settings. Pain medication regimens were divided into mutually exclusive categories based on the combination of all pain medication exposures during follow-up (eg, APAP-hydrocodone, APAP only). We described pain medication regimens overall during the full follow-up period and during 5 distinct time intervals: 0–15 days, 16–30 days, 31–45 days, 46–60 days, and 61–100 days after SNF admission. We anticipated that individuals’ pain control needs would change over time, resulting in corresponding changes in medication regimens over time. Because we expected that changes would occur more frequently in the beginning of the recovery process, we chose to examine shorter intervals during that time period that would align with typical days’ supply values (ie, initial opioid prescriptions are typically <14 days, leading to 15-day intervals until later during follow-up, in which prescriptions would likely be 30–60 days). A person was considered to be exposed to a medication within an interval if the days’ supply overlapped with that interval. Co-prescribing of gabapentinoids and opioids was defined as dispensing of a gabapentinoid and dispensing of an opioid occurring in the same interval.

### Patient Characteristics

We constructed measures of patients’ clinical and demographic characteristics and characteristics of their hip fracture hospitalization using information in the 365 days of their hospital admission date and their hip fracture hospitalization claim. Clinical characteristics included chronic conditions categorized using the Healthcare Cost and Utilization Project Clinical Classifications Software (HCUP),^24^ a claims-based frailty index applied to Medicare claims,^25^ and pain medication receipt before the eligible SNF stay measured using both Omnicare records and Medicare Part D claims. Demographic variables included age, sex, race/ethnicity (measured via the Research Triangle Institute algorithm^26^), and dual enrollment in Medicare and Medicaid. Characteristics of hip fracture hospitalization included an indicator of whether the hospitalization began with an emergency department visit, length of hospital stay, occurrence of selected complications, and type of fracture management procedure.

### Statistical Analysis

To better understand generalizability of the Omnicare cohort to the overall Medicare fee-for-service population with a hip fracture hospitalization discharged to an SNF, we compared characteristics between the linked and full eligible cohort and calculated the standardized mean difference (SMD) of key characteristics between the linked and nonlinked groups. An SMD of greater than 0.10 (10%) is considered to indicate a meaningful difference between groups.^27^ We estimated the overall proportion of patients dispensed any pain medication and specific pain medication regimens by dividing the number of individuals with a relevant medication dispensing at any point during follow-up by the number in the entire cohort. In estimating the prevalence of medication dispensings within each time interval (eg, days 0–15 after SNF admission), we used the number of individuals with a relevant medication dispensing during the period as the numerator and the full cohort number of individuals alive and uncensored at the beginning of the follow-up period as the denominator. We examined the most prevalent pain medication regimens in days 0–15 after SNF admission stratified by baseline pain severity/frequency to determine whether regimens varied by pain level. We assessed pain medication dispensings by sex and race/ethnicity to identify potential differences in pain management across demographic groups. Finally, to assess the prevalence of co-prescribing of gabapentinoids and opioids, we evaluated the frequency of concurrent dispensing in the first 30 days of follow-up, when we hypothesized risk of co-prescribing would be highest. All analyses were performed using SAS, version 9.4 (SAS Institute).

## Results

### Description of Study Population

Between 2012 and 2018, we identified 1,569,299 Medicare beneficiaries with a hip fracture hospitalization (Figure 1), among whom 423,347 (27.0%) met the criteria for study inclusion before data linkage. Of these, 88,433 (20.8%) were among those linked to Omnicare data and included in the final population for analysis [76.8% female, mean age = 84.8 (SD = 8.1 years), 93.3% non-Hispanic White, 28.1% dual Medicare-Medicaid enrollment; Table 1]. Approximately 40% of individuals had documented opioid use in Part D claims or Omnicare records in the 12 months before their hip fracture hospitalization. The Omnicare-linked cohort had characteristics similar to the full eligible cohort, but Omnicare-linked individuals had a higher prevalence of some chronic conditions, including anemia, hypertension, and dementia (Supplementary Table 3).

### Pain Medication Dispensing Following SNF Admission

**Fig. 2.**
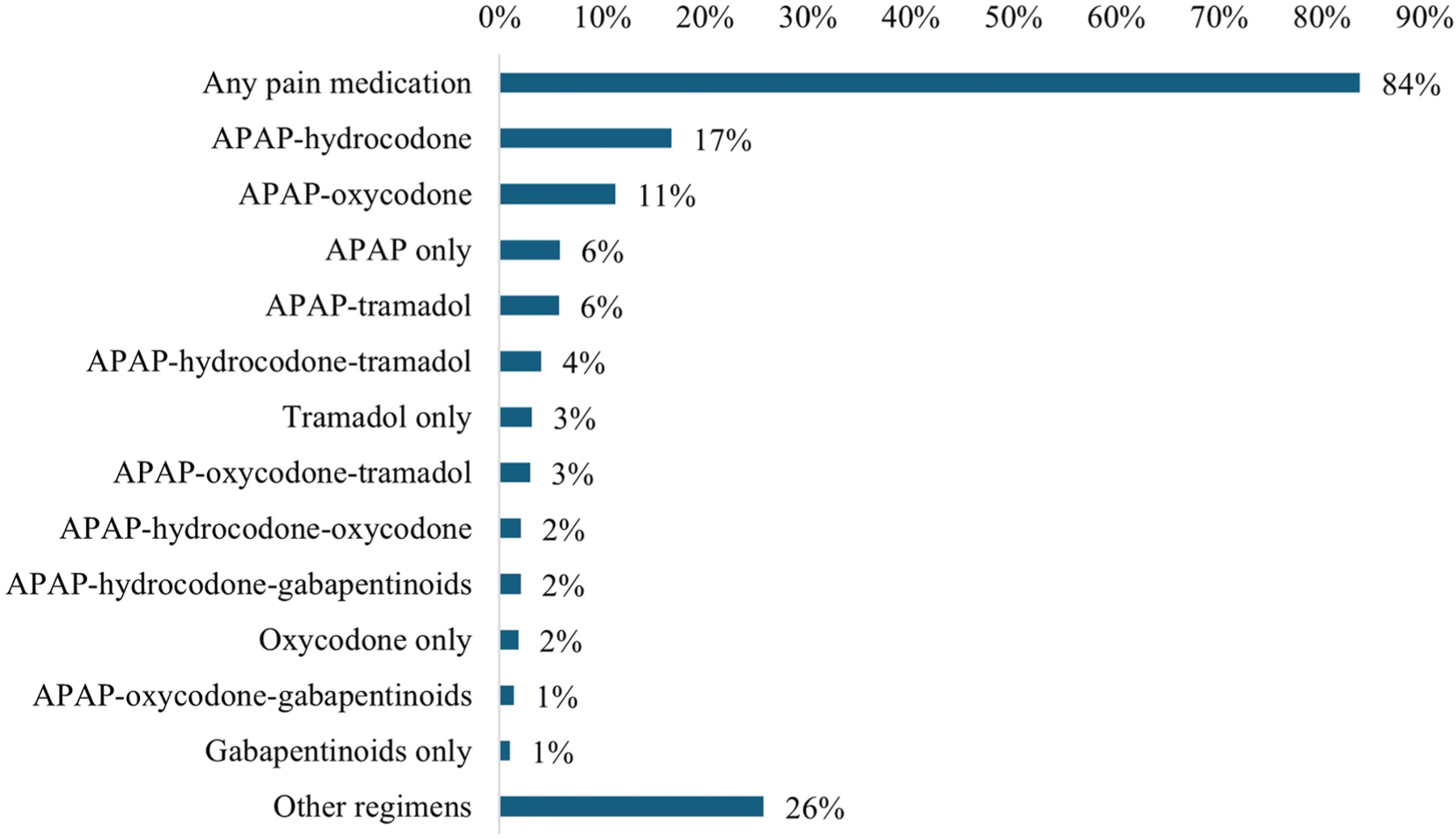
Prevalence of pain medication dispensing up to 100 days following hip fracture hospitalization among older adult Medicare beneficiaries discharged to SNFs. APAP, acetaminophen.

Figure 2 presents the prevalence of any pain medication dispensing and dispensing of specific pain medication regimens across the full follow-up period. Overall, 74,028 individuals (83.7% of the study population) received pain medications at any point during the 100-day follow-up. Of these, 63,427 individuals (85.7%) had pain medication dispensings in Omnicare records, and 36,407 (49.2%) had pain medication dispensings in Part D claims. The most common regimen was a combination of APAP and hydrocodone (16.7% prevalence across the follow-up period), followed by APAP-oxycodone (11.2%), APAP only (5.9%), and APAP-tramadol (5.8%). The array of observed pain medication regimens was diverse, with 28.1% of patients receiving a regimen that had less than 2% total prevalence across the follow-up period [eg, APAP-hydrocodone-morphine (0.89%); APAP-hydrocodone-tramadol-gabapentinoids (0.82%)].

### Changes in Pain Management Over Time After SNF Admission

In the first 15 days after SNF admission, 67.2% of individuals had at least 1 pain medication dispensing (Supplementary Table 4). This prevalence decreased to 38.9% in days 16 to 30 and continued to consistently decline in subsequent periods before increasing in the final period (26.1% prevalence in days 46–60 vs 36.5% in days 61–100). Among the individuals who had no pain medication dispensing in days 0–15 (n = 29,000), 50.3% received a pain medication in a future interval. The most prevalent pain medication regimens in days 0 to 15 following SNF admission were similar among patients who reported no pain, mild/infrequent pain, and severe/frequent pain (Supplementary Table 5).

**Fig. 3.**
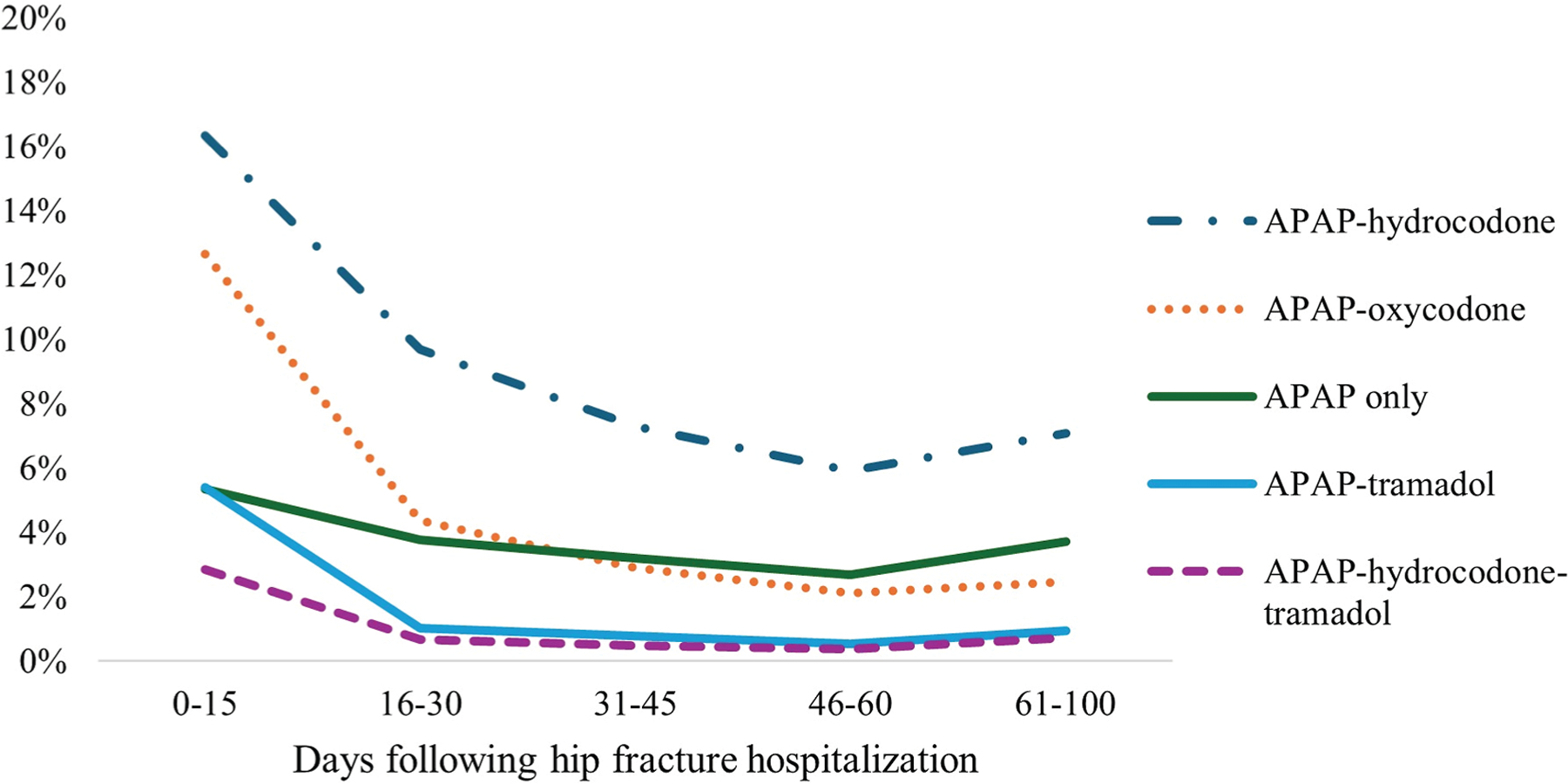
Prevalence of dispensing of selected pain medication regimens over time following hip fracture hospitalization among older adult Medicare beneficiaries discharged to SNFs (denominator N = 88,433). APAP, acetaminophen.

Figure 3 presents the prevalence of the most common pain medication regimens by time interval following SNF admission. Dispensing of opioid-based regimens generally declined by more than 50% from the first 15 days after SNF admission relative to days 31 to 45 after admission, with greater declines observed for most oxycodone-based regimens as compared with hydrocodone-based regimens. However, dispensing of several opioid-based regimens then increased by days 61 to 100 following SNF admission (eg, 5.9% prevalence in days 46–60 vs 7.1% prevalence in days 61–100 for APAP-hydrocodone). In contrast to most opioid-based regimens, dispensing of regimens containing only tramadol or gabapentinoids increased over time (ie, tramadol-only increased from 1.9% in days 0–15 to 4.2% in days 16–30; gabapentinoids from 0.3% in days 0–15 to 3.0% in days 61–100; Supplementary Table 4).

### Pain Medication Dispensing by Sex and Race/Ethnicity

The prevalence of any pain medication dispensing during follow-up was similar between sex subgroups, although slightly higher for women vs men (84.2% vs 82.2%; Supplementary Table 6). Trends in the prevalence of any pain medication dispensing were also similar over time when stratified by sex. No substantial differences in dispensing of specific pain medication regimens by sex were observed (Supplementary Table 7).

The prevalence of any pain medication dispensing during follow-up was similar between race/ethnicity subgroups (eg, 83.8% among non-Hispanic White individuals vs 82.8% among Black/African American individuals and approximately 82% among both Asian/Pacific Islander and Hispanic individuals; Supplementary Table 8). As shown in Supplementary Table 9, the prevalence of APAP-hydrocodone receipt was higher among Asian/Pacific Islander individuals (21.1%) than among non-Hispanic White individuals (16.7%) and Black/African American individuals (16.4%), and a lower proportion of Asian/Pacific Islander individuals received APAP-oxycodone (9.8%) as compared with other race/ethnicity groups (eg, 11.2% among non-Hispanic White individuals).

### Gabapentinoid and Opioid Co-prescribing

A total of 6671 individuals had a gabapentinoid dispensing in the first 15 days after SNF admission, of whom 18.8% had not received a gabapentinoid before hip fracture hospitalization (Table 2). In days 16 to 30 after SNF admission, 4805 individuals had a gabapentinoid dispensing, and 20.8% of these individuals had no gabapentinoid prescription before hip fracture hospitalization. Among individuals dispensed a gabapentinoid in days 0 to 15 after SNF admission, 91.0% were co-prescribed an opioid during the same period, and the proportion with opioid co-prescribing was similar between those without and with gabapentinoid receipt pre-hospitalization (91.3% vs 90.9%, respectively). Compared with days 0 to 15, the prevalence of opioid co-prescribing was lower among individuals dispensed a gabapentinoid in days 16 to 30 after SNF admission (58.2%) and again did not differ substantially by pre-hospitalization gabapentinoid receipt.

## Discussion

In this national cohort study using a novel data linkage between Medicare claims and dispensing data from a large commercial SNF pharmacy, we found that nearly 84% of older adult Medicare beneficiaries discharged to an eligible SNF had a pain medication dispensing up to 100 days following a hip fracture hospitalization. Pain medication regimens varied markedly across individuals and over time. Guideline-concordant multimodal medication regimens were most common, but we also observed a high prevalence of potentially problematic gabapentinoid-opioid co-prescribing among older adults who received a gabapentinoid in the first 15 days after SNF admission (91%). Our study cohort had characteristics similar to cohorts in prior studies of individuals discharged to SNFs after hip fracture.^28–30^

Consistent with a previous study of analgesic regimens administered in SNF PAC after hip fracture in a smaller sample from a single SNF chain, we found that regimens combining APAP with oxycodone or hydrocodone were most common, followed by APAP monotherapy.^31^ This finding suggests that clinicians most commonly prescribe medication regimens for hip fracture pain management in SNFs that comply with the Centers for Disease Control and Prevention’s Clinical Practice Guideline for Prescribing Opioids for Pain, the American Academy of Orthopaedic Surgeons Evidence-Based Guideline on Management of Hip Fractures in the Elderly, and the International Geriatric Fracture Society Consensus Statement, all of which recommend multimodal pain control.^9,10,12^ Given limited evidence on the effectiveness of specific opioid-sparing regimens after hospital discharge,^15,19^ our findings also highlight the need for comparative effectiveness studies of combined APAP-opioid regimens and APAP monotherapy to guide analgesic prescribing in SNFs.

The declining prevalence of most opioid-containing regimens after SNF admission that we observed could suggest that clinicians seek to mitigate the risks of opioid use by limiting opioid prescribing to the immediate postfracture period when opioids are most necessary. However, we saw an increase in dispensing of any pain medication, and particularly of opioid-containing regimens, in the 61 to 100 days following SNF admission. This pattern may reflect persistent or recurrent pain during later stages of recovery among older adults who were unable to rehabilitate well and are making a transition to long-term care in the nursing facility. Alternatively, as-needed use of initial opioid dispensings may have extended true exposures beyond the recorded days’ supply (eg, a 30 days’ supply may extend to 45 or 60 days), inflating exposures due to refills after day 60.^30^ Some of this increase may also reflect discharge from the SNF, resulting in a new prescription dispensing for at-home use. Studies incorporating patient-reported outcomes could provide insights into whether clinicians may be undertreating pain for some patients later in the recovery period.

We did not observe substantial differences in overall pain medication dispensing or changes in dispensing over time by sex or race and ethnicity. However, notable differences emerged in the pain medication regimens used across race/ethnicity subgroups, with greater APAP-hydrocodone dispensing and lower APAP-oxycodone dispensing among Asian/Pacific Islander individuals compared with other race/ethnicity groups. This finding builds on evidence of differences in opioid dosages by race/ethnicity among older adults transitioning from institutional PAC settings to the community following hip fracture.^4^ Future research could examine factors that explain these differences in medication regimens and assess whether these differences result in disparities in clinical outcomes.

Importantly, our study provides evidence that gabapentinoid-opioid co-prescribing was nearly ubiquitous among older adults who were dispensed a gabapentinoid immediately following SNF admission. Co-prescribing of opioids and gabapentinoids in SNFs may conflict with the US Food and Drug Administration’s December 2019 safety communication about the risk of serious breathing problems when gabapentinoids are used in combination with opioids and other central nervous system depressants, particularly among older adults.^21^ We also found that the gabapentinoids-only regimen was one of a limited number in our study with a prevalence that increased over time following SNF admission. Although prescribers may be using gabapentinoids as an opioid-sparing strategy,^32,33^ the clinical benefit of gabapentinoids for this purpose is uncertain,^14,15^ posing the risk of inadequate pain control. Careful medication management in SNFs is needed to avoid the risks of both potentially problematic medication combinations, like gabapentinoid-opioid co-prescribing,^16–18^ and ineffective pain management.^5–7^

Our findings must be interpreted considering several limitations. First, our findings may not be generalizable to older adults hospitalized for hip fracture who were not continuously enrolled in fee-for-service Medicare and Part D or were not discharged to an SNF. In addition, given that pain medication exposures were ascertained from 2 different data sources, we did not estimate doses to avoid errors in cases of overlap between Part D claims and Omnicare records. We ascertained medication exposures in an interval based on dispensing date and days’ supply variables but were unable to measure actual medication administrations during an interval. Finally, we observe dispensing of prescription and over-the-counter pain medications but cannot confirm whether patients used all dispensed medications. Despite these limitations, our study provides novel evidence describing pain medication dispensing among older adults discharged to an SNF following hip fracture hospitalization, a setting where medication prescribing is not typically observable in population-based US studies.

## Conclusions and Implications

Among older adults discharged to an SNF following hip fracture hospitalization, most received some form of pain medication after SNF admission. Many different pain medication regimens were used, but guideline-concordant multimodal opioid-containing regimens were most common. The prevalence of opioid co-prescribing among individuals receiving a gabapentinoid immediately after SNF admission was high. Careful medication management in SNF PAC is needed to ensure adequate pain control throughout the hip fracture recovery period while minimizing potentially harmful analgesic combinations, like gabapentinoids and opioids.

## Supplementary Material

Supplementary Material

Supplementary data related to this article can be found online at https://doi.org/10.1016/j.jamda.2025.105922.

## Figures and Tables

**Table 1:** Characteristics of Older Adult Medicare Beneficiaries Discharged to SNFs After Hip Fracture Hospitalization and Who Were Linked to Omnicare Pharmacy Records, 2012–2018

	N = 88,433
Demographic characteristics	
Age, y, mean (SD)	84.83 (8.05)
Sex	
Female	67,869 (76.75)
Male	20,564 (23.25)
Race/ethnicity	
Non-Hispanic White	82,509 (93.30)
Non-Hispanic Black	2082 (2.35)
Hispanic	1209 (1.37)
Other	2341 (2.65)
Unknown	292 (0.33)
Dual Medicare/Medicaid enrollment	24,808 (28.05)
Clinical characteristics	
Frailty index*	
Robust	16,356 (18.50)
Prefrail	62,385 (70.54)
Mildly-to-severely frail	9692 (10.96)
Gagne comorbidity score, mean (SD)	3.32 (2.38)
Pain severity/frequency^†^	
None	17,249 (19.51)
Mild/infrequent	40,356 (45.63)
Severe/frequent	29,704 (33.59)
Missing	1124 (1.27)
Medication use before hip fracture hospitalization^‡^	
Opioids	36,050 (40.76)
NSAIDs	8662 (9.79)
Gabapentinoids	11,837 (13.39)
Benzodiazepines	21,000 (23.75)
Hip fracture hospitalization characteristics	
Admission from emergency department	14,309 (16.18)
Length of stay, d, mean (SD)	5.30 (2.92)
Fracture management^§^	
Partial or total joint replacement	27,491 (31.09)
Any internal fixation or external fixation using open or percutaneous approach	49,129 (55.56)
Other surgical management	197 (0.22)
Nonsurgical management	45,083 (50.98)

NSAIDs, non-steroidal anti-inflammatory drugs; SD, standard deviation. Reports n (%), unless otherwise stated.

* Measured using the claims-based frailty index and categorized as <0.15 (robust), 0.15–0.24 (prefrail), ≥0.25 (mildly-to-severely frail).

† Pain severity/frequency was measured using the MDS clinical assessment with an assessment date closest to the date of SNF admission.

‡ Medication use was defined as at least 1 month of medication use in the 12 months before the hip fracture hospitalization using both Part D claims data and Omnicare drug claims data.

§ Fracture management was ascertained from International Classification of Diseases (ICD)-9 and ICD-10 procedure codes documented during the hip fracture hospitalization. Participants could be represented in more than 1 fracture management category.

**Table 2:** Prevalence of Opioid Co-prescribing Among Older Adult Medicare Beneficiaries Who Received a Gabapentinoid Following Hip Fracture Hospitalization and Discharge to SNFs

	Overall	By Baseline Gabapentinoid Use	
		Received Gabapentinoid Before Hip Fracture	Did Not Receive Gabapentinoid Before Hip Fracture
Prescribed gabapentinoid 0–15 days posthospitalization	n = 6671	n = 5417	n = 1254
Opioid co-prescribed,* n (%)	6069 (90.98%)	4924 (90.90%)	1145 (91.31%)
Prescribed gabapentinoid 16–30 days posthospitalization	n = 4805	n = 3807	n = 998
Opioid co-prescribed,* n (%)	2798 (58.23%)	2236 (58.73%)	562 (56.31%)

* Co-prescribing occurs when a beneficiary has both an opioid and gabapentinoid dispensing during the same time interval (eg, individuals with gabapentin dispensed 0–15 days after hip fracture who have an opioid dispensed 0–15 days after hip fracture).
