# Supplementary material for: Longitudinal Pain Medication Use Among US Older Adults After a Hip Fracture
Source: J Am Med Dir Assoc. Author manuscript; Available in PMC 2025 Dec 2. (PMC12666629; doi:10.1016/j.jamda.2025.105922)
Supplement: Supplementary Material [file NIHMS2122190-supplement-Supplementary_Material.docx]

**SUPPLEMENTARY MATERIALS**

**Title:** Longitudinal Pain Medication Use Among U.S. Older Adults after a Hip Fracture

**Authors:** Lexie R. Grove, PhD, MSPH, Andrew R. Zullo, PharmD, PhD, Daniel A. Harris, PhD, MPH, et al.

**Supplementary Table S1.** List of diagnosis codes used to identify hip fracture hospitalization.

**Supplementary Table S2.** List of eligible active ingredients by drug class.

**Supplementary Table S3.** Comparison of eligible population of older adult Medicare beneficiaries discharged to skilled nursing facilities after hip fracture hospitalization and subpopulation linked to Omnicare pharmacy records, 2012-2018.

**Supplementary Table S4.** Prevalence of dispensing of selected pain medication regimens by time interval following hip fracture hospitalization among older adult Medicare beneficiaries discharged to skilled nursing facilities, 2012-2018.

**Supplementary Table S5.** Most prevalent pain medication regimens by baseline pain severity/frequency in days 0-15 after hip fracture hospitalization among older adult Medicare beneficiaries discharged to skilled nursing facilities, 2012-2018.

**Supplementary Table S6.** Prevalence of any pain medication dispensing by beneficiary sex over time following hip fracture hospitalization among older adult Medicare beneficiaries discharged to skilled nursing facilities, 2012-2018.

**Supplementary Table S7.** Prevalence of dispensing of selected pain medication regimens by beneficiary sex following hip fracture hospitalization among older adult Medicare beneficiaries discharged to skilled nursing facilities, 2012-2018.

**Supplementary Table S8.** Prevalence of any pain medication dispensing by beneficiary race/ethnicity over time following hip fracture hospitalization among older adult Medicare beneficiaries discharged to skilled nursing facilities, 2012-2018.

**Supplementary Table S9.** Prevalence of dispensing of selected pain medication regimens by beneficiary race/ethnicity following hip fracture hospitalization among older adult Medicare beneficiaries discharged to skilled nursing facilities, 2012-2018.

**Supplementary Table S1.** List of diagnosis codes used to identify hip fracture hospitalization.

| **International Classification of Diseases (ICD) version** | **Code** | **Label** |
| --- | --- | --- |
| ICD-9-CM | 82000 | Closed fracture of intracapsular section of neck of femur, unspecified (82000) |
| ICD-9-CM | 82001 | Closed fracture of epiphysis (separation) (upper) of neck of femur (82001) |
| ICD-9-CM | 82002 | Closed fracture of midcervical section of neck of femur (82002) |
| ICD-9-CM | 82003 | Closed fracture of base of neck of femur (82003) |
| ICD-9-CM | 82009 | Other closed transcervical fracture of neck of femur (82009) |
| ICD-9-CM | 82010 | Open fracture of intracapsular section of neck of femur, unspecified (82010) |
| ICD-9-CM | 82011 | Open fracture of epiphysis(separation) (upper) of neck of femur (82011) |
| ICD-9-CM | 82012 | Open fracture of midcervical section of neck of femur (82012) |
| ICD-9-CM | 82013 | Open fracture of base of neck of femur (82013) |
| ICD-9-CM | 82019 | Other open transcervical fracture of neck of femur (82019) |
| ICD-9-CM | 82020 | Closed fracture of trochanteric section of neck of femur (82020) |
| ICD-9-CM | 82021 | Closed fracture of intertrochanteric section of neck of femur (82021) |
| ICD-9-CM | 82022 | Closed fracture of subtrochanteric section of neck of femur (82022) |
| ICD-9-CM | 82030 | Open fracture of trochanteric section of neck of femur, unspecified (82030) |
| ICD-9-CM | 82031 | Open fracture of intertrochanteric section of neck of femur (82031) |
| ICD-9-CM | 82032 | Open fracture of subtrochanteric section of neck of femur (82032) |
| ICD-9-CM | 8208 | Closed fracture of unspecified part of neck of femur (8208) |
| ICD-9-CM | 8209 | Open fracture of unspecified part of neck of femur (8209) |
| ICD-10-CM | S72001 | Fracture of unspecified part of neck of right femur (S72001) |
| ICD-10-CM | S72002 | Fracture of unspecified part of neck of left femur (S72002) |
| ICD-10-CM | S72009 | Fracture of unspecified part of neck of unspecified femur (S72009) |
| ICD-10-CM | S72011 | Unspecified intracapsular fracture of right femur (S72011) |
| ICD-10-CM | S72012 | Unspecified intracapsular fracture of left femur (S72012) |
| ICD-10-CM | S72019 | Unspecified intracapsular fracture of unspecified femur (S72019) |
| ICD-10-CM | S72021 | Displaced fracture of epiphysis (separation) (upper) of right femur (S72021) |
| ICD-10-CM | S72022 | Displaced fracture of epiphysis (separation) (upper) of left femur (S72022) |
| ICD-10-CM | S72023 | Displaced fracture of epiphysis (separation) (upper) of unspecified femur (S72023) |
| ICD-10-CM | S72024 | Nondisplaced fracture of epiphysis (separation) (upper) of right femur (S72024) |
| ICD-10-CM | S72025 | Nondisplaced fracture of epiphysis (separation) (upper) of left femur (S72025) |
| ICD-10-CM | S72031 | Displaced midcervical fracture of right femur (S72031) |
| ICD-10-CM | S72032 | Displaced midcervical fracture of left femur (S72032) |
| ICD-10-CM | S72033 | Displaced midcervical fracture of unspecified femur |
| ICD-10-CM | S72034 | Nondisplaced midcervical fracture of right femur (S72034) |
| ICD-10-CM | S72035 | Nondisplaced midcervical fracture of left femur (S72035) |
| ICD-10-CM | S72036 | Nondisplaced midcervical fracture of unspecified femur (S72036) |
| ICD-10-CM | S72041 | Displaced fracture of base of neck of right femur (S72041) |
| ICD-10-CM | S72042 | Displaced fracture of base of neck of left femur (S72042) |
| ICD-10-CM | S72043 | Displaced fracture of base of neck of unspecified femur (S72043) |
| ICD-10-CM | S72044 | Nondisplaced fracture of base of neck of right femur (S72044) |
| ICD-10-CM | S72045 | Nondisplaced fracture of base of neck of left femur (S72045) |
| ICD-10-CM | S72046 | Nondisplaced fracture of base of neck of unspecified femur (S72046) |
| ICD-10-CM | S72051 | Unspecified fracture of head of right femur (S72051) |
| ICD-10-CM | S72052 | Unspecified fracture of head of left femur (S72052) |
| ICD-10-CM | S72059 | Unspecified fracture of head of unspecified femur (S72059) |
| ICD-10-CM | S72061 | Displaced articular fracture of head of right femur (S72061) |
| ICD-10-CM | S72062 | Displaced articular fracture of head of left femur (S72062) |
| ICD-10-CM | S72064 | Nondisplaced articular fracture of head of right femur (S72064) |
| ICD-10-CM | S72065 | Nondisplaced articular fracture of head of left femur (S72065) |
| ICD-10-CM | S72066 | Nondisplaced articular fracture of head of unspecified femur (S72066) |
| ICD-10-CM | S72091 | Other fracture of head and neck of right femur (S72091) |
| ICD-10-CM | S72092 | Other fracture of head and neck of left femur (S72092) |
| ICD-10-CM | S72099 | Other fracture of head and neck of unspecified femur (S72099) |
| ICD-10-CM | S72101 | Unspecified trochanteric fracture of right femur (S72101) |
| ICD-10-CM | S72102 | Unspecified trochanteric fracture of left femur (S72102) |
| ICD-10-CM | S72109 | Unspecified trochanteric fracture of unspecified femur (S72109) |
| ICD-10-CM | S72111 | Displaced fracture of greater trochanter of right femur (S72111) |
| ICD-10-CM | S72112 | Displaced fracture of greater trochanter of left femur (S72112) |
| ICD-10-CM | S72113 | Displaced fracture of greater trochanter of unspecified femur (S72113) |
| ICD-10-CM | S72114 | Nondisplaced fracture of greater trochanter of right femur (S72114) |
| ICD-10-CM | S72115 | Nondisplaced fracture of greater trochanter of left femur (S72115) |
| ICD-10-CM | S72116 | Nondisplaced fracture of greater trochanter of unspecified femur (S72116) |
| ICD-10-CM | S72121 | Displaced fracture of lesser trochanter of right femur (S72121) |
| ICD-10-CM | S72122 | Displaced fracture of lesser trochanter of left femur (S72122) |
| ICD-10-CM | S72123 | Displaced fracture of lesser trochanter of unspecified femur (S72123) |
| ICD-10-CM | S72124 | Nondisplaced fracture of lesser trochanter of right femur (S72124) |
| ICD-10-CM | S72125 | Nondisplaced fracture of lesser trochanter of left femur (S72125) |
| ICD-10-CM | S72126 | Nondisplaced fracture of lesser trochanter of unspecified femur (S72126) |
| ICD-10-CM | S72131 | Displaced apophyseal fracture of right femur (S72131) |
| ICD-10-CM | S72132 | Displaced apophyseal fracture of left femur (S72132) |
| ICD-10-CM | S72133 | Displaced apophyseal fracture of unspecified femur (S72133) |
| ICD-10-CM | S72134 | Nondisplaced apophyseal fracture of right femur (S72134) |
| ICD-10-CM | S72135 | Nondisplaced apophyseal fracture of left femur (S72135) |
| ICD-10-CM | S72141 | Displaced intertrochanteric fracture of right femur (S72141) |
| ICD-10-CM | S72142 | Displaced intertrochanteric fracture of left femur (S72142) |
| ICD-10-CM | S72143 | Displaced intertrochanteric fracture of unspecified femur (S72143) |
| ICD-10-CM | S72144 | Nondisplaced intertrochanteric fracture of right femur (S72144) |
| ICD-10-CM | S72145 | Nondisplaced intertrochanteric fracture of left femur (S72145) |
| ICD-10-CM | S72146 | Nondisplaced intertrochanteric fracture of unspecified femur (S72146) |
| ICD-10-CM | S7221 | Displaced subtrochanteric fracture of right femur (S7221) |
| ICD-10-CM | S7222 | Displaced subtrochanteric fracture of left femur (S7222) |
| ICD-10-CM | S7223 | Displaced subtrochanteric fracture of unspecified femur (S7223) |
| ICD-10-CM | S7224 | Nondisplaced subtrochanteric fracture of right femur (S7224) |
| ICD-10-CM | S7225 | Nondisplaced subtrochanteric fracture of left femur (S7225) |
| ICD-10-CM | S7226 | Nondisplaced subtrochanteric fracture of unspecified femur (S7226) |

**Supplementary Table S2.** List of eligible active ingredients by drug class.

|  | **Eligible active ingredients*** |
| --- | --- |
| **Acetaminophen** | acetaminophen |
| **Opioids** | tramadol  oxycodone  hydrocodone  fentanyl  morphine  hydromorphone  methadone  buprenorphine  tapentadol  oxymorphone  codeine  meperidine  levorphanol  butorphanol |
| **NSAIDs** | ibuprofen  celecoxib  naproxen  diclofenac  nabumetone  etodolac |
| **Gabapentinoids** | gabapentin  pregabalin |
| **Benzodiazepines** | alprazolam  estazolam  lorazepam  midazolam  remimazolam  oxazepam  temazepam  triazolam  chlordiazepoxide  clonazepam  clorazepate  diazepam  flurazepam  quazepam |
| **Muscle relaxants** | cyclobenzaprine  baclofen |

*Any product containing an eligible active ingredient was included, including combination products.

**Supplementary Table S3.** Comparison of eligible population of older adult Medicare beneficiaries discharged to skilled nursing facilities after hip fracture hospitalization and subpopulation linked to Omnicare pharmacy records, 2012-2018.

|  | **Eligible population**  **(n= 423,347)** | **Omnicare-linked**  **(n = 88,433)** | **Non-linked (n=334,914)** | **Absolute standardized mean difference**  **(Omnicare-linked vs. non-linked)** |
| --- | --- | --- | --- | --- |
| **Demographic characteristics** |  |  |  |  |
| Age, years (mean, SD) | 83.76 (8.15) | 84.83 (8.05) | 83.48 (8.15) | 0.167 |
| Sex |  |  |  |  |
| Female | 322,011 (76.06) | 67,869 (76.75) | 254,142 (75.88) | 0.020 |
| Male | 101,336 (23.94) | 20,564 (23.25) | 80,772 (24.12) | 0.020 |
| Race/ethnicity |  |  |  |  |
| Non-Hispanic White | 388,748 (91.83) | 82,509 (93.30) | 306,239 (91.44) | 0.076 |
| Non-Hispanic Black | 16,048 (3.79) | 2,082 (2.35) | 13,966 (4.17) | 0.102 |
| Hispanic | 6,450 (1.52) | 1,209 (1.37) | 5,241 (1.56) | 0.015 |
| Other | 11,038 (2.61) | 2,341 (2.65) | 8,697 (2.60) | 0.000 |
| Unknown | 1,063 (0.25) | 292 (0.33) | 771 (0.23) | 0.000 |
| Dual Medicare/Medicaid enrollment | 123,662 (29.21) | 24,808 (28.05) | 98,854 (29.52) | 0.032 |
| **Year of hip fracture** |  |  |  | 0.148 |
| 2012 | 54,452 (12.86) | 12,413 (14.04) | 42,039 (12.55) |  |
| 2013 | 57,482 (13.58) | 12,924 (14.61) | 44,558 (13.30) |  |
| 2014 | 63,700 (15.05) | 14,845 (16.79) | 48,855 (14.59) |  |
| 2015 | 63,429 (14.98) | 13,801 (15.61) | 49,628 (14.82) |  |
| 2016 | 60,397 (14.27) | 12,696 (14.36) | 47,701 (14.24) |  |
| 2017 | 62,805 (14.84) | 11,823 (13.37) | 50,982 (15.22) |  |
| 2018 | 61,082 (14.43) | 9,931 (11.23) | 51,151 (15.27) |  |
| **Clinical characteristics*** |  |  |  |  |
| Acute myocardial infarction | 3,589 (0.85) | 829 (0.94) | 2,760 (0.82) | 0.012 |
| Acute phlebitis, thrombophlebitis, or thromboembolism | 1,730 (0.41) | 327 (0.37) | 1,403 (0.42) | 0.008 |
| Anemia | 143,277 (33.84) | 32,897 (37.20) | 110,380 (32.96) | 0.089 |
| Asthma | 10,579 (2.50) | 2,365 (2.67) | 8,214 (2.45) | 0.014 |
| Cancer | 40,772 (9.63) | 9,363 (10.59) | 31,409 (9.38) | 0.040 |
| Cardiac dysrhythmias | 68,536 (16.19) | 15,962 (18.05) | 52,574 (15.70) | 0.063 |
| Cerebrovascular disease | 16,052 (3.79) | 3,650 (4.13) | 12,402 (3.70) | 0.022 |
| Chronic kidney disease | 33,261 (7.86) | 6,652 (7.52) | 26,609 (7.95) | 0.016 |
| Chronic obstructive pulmonary disease and bronchiectasis | 39,628 (9.36) | 8,562 (9.68) | 31,066 (9.28) | 0.014 |
| Coronary atherosclerosis and other heart diseases | 26,337 (6.22) | 5,256 (5.94) | 21,081 (6.29) | 0.015 |
| Dementia, delirium, and other cognitive disorders | 83,212 (19.66) | 20,038 (22.66) | 63,174 (18.86) | 0.094 |
| Diabetes mellitus | 106,761 (25.22) | 21,521 (24.34) | 85,240 (25.45) | 0.026 |
| Gout and other crystal arthropathies | 7,036 (1.66) | 1,600 (1.81) | 5,436 (1.62) | 0.014 |
| Heart valve disorders | 29,093 (6.87) | 7,084 (8.01) | 22,009 (6.57) | 0.055 |
| Hypertension | 173,440 (40.97) | 39,301 (44.44) | 134,139 (40.05) | 0.089 |
| Liver disease | 4,741 (1.12) | 1,036 (1.17) | 3,705 (1.11) | 0.006 |
| Low back pain | 2,817 (0.67) | 581 (0.66) | 2,236 (0.67) | 0.001 |
| Other musculoskeletal pain | 9,405 (2.22) | 1,804 (2.04) | 7,601 (2.27) | 0.016 |
| Obesity | 9,979 (2.36) | 2,099 (2.37) | 7,880 (2.35) | 0.001 |
| Opioid related disorders | 5,504 (1.30) | 1,094 (1.24) | 4,410 (1.32) | 0.007 |
| Osteoarthritis | 39,377 (9.30) | 8,745 (9.89) | 30,632 (9.15) | 0.025 |
| Peripheral and visceral atherosclerosis | 13,190 (3.12) | 2,684 (3.04) | 10,506 (3.14) | 0.006 |
| Phlebitis, thrombophlebitis, and thromboembolism | 11,083 (2.62) | 2,576 (2.91) | 8,507 (2.54) | 0.023 |
| Pulmonary heart disease | 15,464 (3.65) | 3,663 (4.14) | 11,801 (3.52) | 0.032 |
| Rheumatoid arthritis and related disease | 6,179 (1.46) | 1,168 (1.32) | 5,011 (1.50) | 0.015 |
| Schizophrenia and other psychotic disorders | 6,281 (1.48) | 1,393 (1.58) | 4,888 (1.46) | 0.009 |
| Thyroid disorder | 56,267 (13.29) | 13,020 (14.72) | 43,247 (12.91) | 0.052 |
| Frailty index**^†^** |  |  |  | 0.116 |
| Robust | 90,007 (21.26) | 16,356 (18.50) | 73,651 (21.99) | 0.087 |
| Prefrail | 292,117 (69.00) | 62,385 (70.54) | 229,732 (68.59) | 0.042 |
| Mildly-to-severely frail | 41,223 (9.74) | 9,692 (10.96) | 31,531 (9.41) | 0.051 |
| Gagne comorbidity score (mean, SD) | 3.21 (2.36) | 3.32 (2.38) | 3.18 (2.35) |  |
| **Hip fracture hospitalization characteristics** |  |  |  |  |
| Admission from emergency department | 64,688 (15.28) | 14,309 (16.18) | 50,379 (15.04) | 0.031 |
| Length of stay, days (mean, SD) | 5.27 (2.99) | 5.30 (2.92) | 5.26 (3.01) | 0.014 |
| Hospital complications |  |  |  |  |
| Urinary tract infections | 85,262 (20.14) | 18,812 (21.27) | 66,450 (19.84) | 0.035 |
| Pressure ulcer of skin | 4,722 (1.12) | 1,002 (1.13) | 3,720 (1.11) | 0.002 |
| Pneumonia | 16,713 (3.95) | 3,490 (3.95) | 13,223 (3.95) | 0.000 |
| **Fracture management^‡^** |  |  |  |  |
| Partial or total joint replacement | 133,697 (31.58) | 27,491 (31.09) | 106,206 (31.71) | 0.013 |
| Any internal fixation or external fixation using open or percutaneous approach | 228,016 (53.86) | 49,129 (55.56) | 178,887 (53.41) | 0.043 |
| Other surgical management | 983 (0.23) | 197 (0.22) | 786 (0.23) | 0.003 |
| Non-surgical management | 214,702 (50.72) | 45,083 (50.98) | 169,619 (50.65) | 0.007 |

**Abbreviations:** SD, standard deviation; NSAIDs, non-steroidal anti-inflammatory drugs.

**Notes:** Reports number (%), unless otherwise stated.

*Represents the conditions documented on the hip fracture hospitalization claim.

**^†^**Measured using the Claims-based Frailty Index and categorized as: <0.15 (robust), 0.15-0.24 (prefrail), ≥0.25 (mildly-to-severely frail).

^‡^Fracture management was ascertained from ICD-9 and ICD-10 procedure codes documented during the hip fracture hospitalization. Participants could be represented in more than one fracture management category.

**Supplementary Table S4.** Prevalence of dispensing of selected pain medication regimens by time interval following hip fracture hospitalization among older adult Medicare beneficiaries discharged to skilled nursing facilities, 2012-2018.

|  | **Time interval following hip fracture hospitalization** | | | | |
| --- | --- | --- | --- | --- | --- |
|  | *0-15 days* | *16-30 days* | *31-45 days* | *46-60 days* | *61-100 days* |
| Any pain medication | 67.21% | 38.92% | 30.97% | 26.11% | 36.45% |
| APAP-hydrocodone | 16.34% | 9.70% | 7.31% | 5.92% | 7.08% |
| APAP-oxycodone | 12.64% | 4.36% | 2.91% | 2.11% | 2.46% |
| APAP only | 5.35% | 3.77% | 3.19% | 2.70% | 3.70% |
| APAP-tramadol | 5.41% | 1.01% | 0.77% | 0.54% | 0.94% |
| APAP-hydrocodone-tramadol | 2.86% | 0.68% | 0.49% | 0.38% | 0.72% |
| Tramadol only | 1.89% | 4.18% | 3.72% | 3.19% | 4.30% |
| APAP-oxycodone-tramadol | 2.19% | 0.34% | 0.19% | 0.15% | 0.26% |
| APAP-hydrocodone-oxycodone | 1.26% | 0.43% | 0.22% | 0.15% | 0.32% |
| APAP-hydrocodone-gabapentinoids | 1.62% | 0.87% | 0.68% | 0.61% | 1.13% |
| Oxycodone only | 1.62% | 2.94% | 2.16% | 1.52% | 1.48% |
| APAP-oxycodone-gabapentinoids | 1.36% | 0.42% | 0.34% | 0.27% | 0.44% |
| Gabapentinoids only | 0.30% | 1.90% | 2.04% | 2.25% | 3.00% |
| Other regimens | 14.37% | 8.31% | 6.94% | 6.32% | 10.62% |

**Abbreviations**: APAP, acetaminophen.

**Supplementary Table S5.** Most prevalent pain medication regimens by baseline pain severity/frequency in days 0-15 after hip fracture hospitalization among older adult Medicare beneficiaries discharged to skilled nursing facilities, 2012-2018.

| **Pain severity/**  **frequency** | **5 most common pain medication regimens (in descending order)** |
| --- | --- |
| None | 1. APAP-hydrocodone 2. APAP-oxycodone 3. APAP 4. APAP-tramadol 5. Tramadol |
| Mild/Infrequent | 1. APAP-hydrocodone 2. APAP-oxycodone 3. APAP-tramadol 4. APAP 5. APAP-hydrocodone-tramadol |
| Severe/  Frequent | 1. APAP-hydrocodone 2. APAP-oxycodone 3. APAP-tramadol 4. APAP-hydrocodone-tramadol 5. APAP |
| Missing | 1. APAP-hydrocodone 2. APAP-oxycodone 3. APAP-tramadol 4. APAP 5. APAP-hydrocodone-tramadol |

**Supplementary Table S6.** Prevalence of any pain medication dispensing by beneficiary sex over time following hip fracture hospitalization among older adult Medicare beneficiaries discharged to skilled nursing facilities, 2012-2018.

|  | **Male (n=20,564)** | **Female**  **(n=67,869)** |
| --- | --- | --- |
| Any time during follow-up | 82.2% | 84.2% |
| *Days following hip fracture hospitalization* |  |  |
| 0-15 | 66.5% | 67.4% |
| 16-30 | 36.2% | 39.8% |
| 31-45 | 28.7% | 31.7% |
| 46-60 | 23.7% | 26.8% |
| 61-100 | 33.0% | 37.5% |

**Supplementary Table S7.** Prevalence of dispensing of selected pain medication regimens by beneficiary sex following hip fracture hospitalization among older adult Medicare beneficiaries discharged to skilled nursing facilities, 2012-2018.

|  | **Male (n=20,564)** | **Female**  **(n=67,869)** |
| --- | --- | --- |
| APAP-hydrocodone | 17.07% | 16.60% |
| APAP-oxycodone | 12.19% | 10.95% |
| APAP only | 6.40% | 5.68% |
| APAP-tramadol | 5.00% | 3.26% |
| APAP-hydrocodone-tramadol | 3.63% | 4.17% |
| Tramadol only | 2.78% | 3.26% |
| APAP-oxycodone-tramadol | 2.64% | 3.03% |
| APAP-hydrocodone-oxycodone | 2.13% | 2.05% |
| APAP-hydrocodone-gabapentinoids | 2.09% | 2.03% |
| Oxycodone only | 1.97% | 1.76% |
| APAP-oxycodone-gabapentinoids | 1.45% | 1.39% |
| Gabapentinoids only | 1.06% | 0.97% |

**Abbreviations**: APAP, acetaminophen.

**Supplementary Table S8.** Prevalence of any pain medication dispensing by beneficiary race/ethnicity over time following hip fracture hospitalization among older adult Medicare beneficiaries discharged to skilled nursing facilities, 2012-2018.

|  | **Non-Hispanic white (n=82,604)** | **Black/**  **African-American (n=2,083)** | **Asian/**  **Pacific Islander (n=1,277)** | **Hispanic (n=1,217)** | **American Indian/**  **Alaska Native (n=325)** | **Other (n=704)** | **Unknown (n=223)** |
| --- | --- | --- | --- | --- | --- | --- | --- |
| Any time during follow-up | 83.8% | 82.8% | 81.6% | 81.7% | 85.9% | 81.5% | 87.0% |
| *Days following hip fracture hospitalization* |  |  |  |  |  |  |  |
| 0-15 | 67.2% | 65.2% | 68.4% | 68.3% | 67.4% | 68.5% | 75.3% |
| 16-30 | 39.1% | 38.2% | 34.0% | 34.8% | 41.2% | 34.5% | 35.9% |
| 31-45 | 31.1% | 30.6% | 28.4% | 28.8% | 30.5% | 27.6% | 31.4% |
| 46-60 | 26.2% | 27.3% | 22.4% | 22.4% | 29.2% | 24.0% | 28.7% |
| 61-100 | 36.5% | 40.3% | 32.1% | 34.0% | 41.9% | 32.8% | 38.1% |

**Supplementary Table S9.** Prevalence of dispensing of selected pain medication regimens by beneficiary race/ethnicity following hip fracture hospitalization among older adult Medicare beneficiaries discharged to skilled nursing facilities, 2012-2018.

|  | **Non-Hispanic white (n=82,604)** | **Black/**  **African-American (n=2,083)** | **Asian/**  **Pacific Islander (n=1,277)** | **Hispanic (n=1,217)** | **American Indian/**  **Alaska Native (n=325)** | **Other (n=704)** | **Unknown (n=223)** |
| --- | --- | --- | --- | --- | --- | --- | --- |
| APAP-hydrocodone | 16.7% | 16.4% | 21.1% | 14.8% | 17.2% | 16.8% | 13.9% |
| APAP-oxycodone | 11.2% | 12.9% | 9.8% | 12.2% | 10.8% | 12.8% | 11.7% |
| APAP only | 5.8% | 6.6% | 6.4% | 6.7% | 4.0% | 7.4% | 7.6% |
| APAP-tramadol | 5.8% | 5.2% | 4.2% | 6.3% | 4.0% | 3.6% | 5.8% |
| APAP-hydrocodone-tramadol | 4.1% | 4.1% | 3.8% | 2.6% | 4.0% | 3.7% | 2.7% |
| Tramadol only | 3.2% | 3.2% | 2.2% | 3.2% | 3.4% | 3.0% | 1.3% |
| APAP-oxycodone-tramadol | 3.0% | 2.9% | 1.6% | 2.4% | 0.9% | 2.8% | 4.0% |
| APAP-hydrocodone-oxycodone | 2.1% | 1.6% | 1.3% | 1.6% | 1.8% | 2.4% | 4.0% |
| APAP-hydrocodone-gabapentinoids | 2.0% | 2.1% | 3.1% | 1.3% | 1.5% | 2.8% | 1.3% |
| Oxycodone only | 1.8% | 1.2% | 1.6% | 1.0% | 2.8% | 1.6% | 1.3% |
| APAP-oxycodone-gabapentinoids | 1.4% | 1.8% | 1.2% | 1.8% | 0.9% | 1.4% | 1.3% |
| Gabapentinoids only | 1.0% | 0.7% | 1.6% | 1.1% | 1.5% | 0.4% | 0.9% |

**Abbreviations**: APAP, acetaminophen.
